# A Sequential Treatment of Keloids: Multiple‐Hole Punch Excision Combined With Superficial Radiation and Drug

**DOI:** 10.1111/jocd.71197

**Published:** 2026-10-01

**Authors:** Zhicheng Gong, Wenwen Jing, Zhenhua Zhang, Wenbin Rao, Weiqi Lei, Junwu Yang, Yong Ai, Xiaohua Tao

**Affiliations:** ^1^ Dermatology Hospital of Jiangxi Province Nanchang People's Republic of China; ^2^ Jiangxi Provincial Clinical Research Center for Skin Diseases Nanchang People's Republic of China; ^3^ Candidate Branch of National Clinical Research Center for Skin Diseases Nanchang People's Republic of China; ^4^ JXHC Key Laboratory of Skin Infection and Immunity Nanchang People's Republic of China; ^5^ The Affiliated Dermatology Hospital of Nanchang University Nanchang People's Republic of China

**Keywords:** betamethasone, fluorouracil, keloids, multi‐hole punch, sequential treatment, superficial radiotherapy

## Abstract

**Background:**

Dense and multiple keloids present significant therapeutic challenges due to their stubborn nature and high recurrence rates. Although various treatment options exist, optimizing current approaches and integrating them into a rational, systematic regimen for keloid management remains a difficult clinical problem.

**Aims:**

This study aims to establish an effective multimodal sequential treatment protocol for patients with dense, multiple keloids.

**Methods:**

A total of 87 patients with 285 keloid lesions were enrolled from September 2023 to April 2024. All cases underwent a sequential treatment: Stage I involved drug‐assisted multiple‐hole punch excision followed by adjuvant superficial radiotherapy; Stage II consisted of periodic intralesional injections of betamethasone and 5‐fluorouracil. Clinical outcomes, recurrence, and adverse events were evaluated at 12‐month follow‐up. The POSAS and VSS scores were recorded at each time point.

**Results:**

At 12 months post‐treatment, marked improvement was achieved in 78 patients (89.66%), with improvement in 7 (8.04%) and ineffective results in 2 (2.30%). The POSAS and VSS scores were recorded before treatment and at follow‐up points at 30, 60, 90, 180, and 360 days. All patients developed varying degrees of local hyperpigmentation within 2 months after Stage I of multi‐hole punch excision. In 80.75% of cases, the hyperpigmentation resolved within 3 months following Stage I, with the longest duration of resolution being 12 months. During Stage I, four cases of localized ulcers and five instances of acute radiation dermatitis appeared. All adverse reactions were effectively alleviated. No other adverse reactions occurred.

**Conclusion:**

This sequential treatment protocol offers a practical, minimally invasive, and effective option for patients with multiple, dense keloids.

## 1 Introduction

Keloid is a pathological healing process characterized by excessive fibroblast proliferation, disrupted collagen homeostasis, and abnormal extracellular matrix deposition in the dermis following cutaneous injury or inflammation. Recent epidemiological data from large‐scale, multicenter surveys in China reveal a worrying trend: The prevalence of keloids has increased by 64%, rising from 9.2% in 2013 to 15.1% in 2018 [1]. Keloids can lead to pain, itching, infection, deformity, and significantly impact patients' appearance and psychological well‐being, often resulting in cosmetic, functional, and emotional disturbances. As a result, patients affected by keloids urgently seek effective treatments [2]. A wide range of therapeutic modalities is currently available, including surgical excision, intralesional steroid injections, radiotherapy, laser therapy, silicone gel sheets, cryotherapy, photodynamic therapy, and pressure therapy. However, monotherapies generally show limited efficacy and are associated with high recurrence rates. This has prompted clinicians to favor multimodal treatment strategies, such as combining surgery with radiotherapy, integrating laser therapy with corticosteroids, and using corticosteroids alongside botulinum toxin or cryotherapy [3, 4, 5]. However, the management of keloids, especially in cases involving dense or multiple lesions, remains highly challenging. High‐dose corticosteroid regimens can result in local complications, such as cutaneous atrophy and telangiectasia, as well as systemic adverse effects, including adrenal suppression and Cushingoid features. Surgical interventions for extensive lesions are technically demanding and entail high costs and prolonged recovery, further highlighting the urgent need for treatment modalities that are both minimally invasive and comprehensive. This clinical necessity has led to a paradigm shift towards minimally invasive, multimodal combination therapies. It is crucial to distinguish evidence‐based combination treatments from indiscriminate polypharmacy. Effective multimodal regimens require temporal optimization, involving the sequential application of complementary therapies that target distinct pathological stages. This precision‐based approach aims to achieve therapeutic synergy—where the combined effect exceeds the sum of individual treatments—while ensuring efficient use of healthcare resources. Our research team has been dedicated to exploring and evaluating novel therapeutic protocols for the management of dense and multiple keloids. From September 2023 to April 2024, we enrolled a total of 87 keloid patients, all of whom were treated with a ‘sequential treatment’ protocol. Preliminary outcomes have demonstrated encouraging efficacy and favorable safety profiles, the details of which are presented in this report.

## 2 Materials and Methods

### 2.1 Clinical Date

A total of 285 keloid lesions from 87 patients were included in this study, which spanned from September 2023 to April 2024.

### 2.2 Inclusion and Exclusion Criteria

Inclusion criteria for this study are as follows: (1) participants aged 16 years or older; (2) clinically confirmed keloids that have persisted for more than six months; (3) presence of ≥ ≥ 2 lesions per patient with maximum thickness ≤ 5 mm; and (4) patients who are capable of receiving treatment and adhering to follow‐up schedules. The exclusion criteria include: (1) active infections or functional contractures associated with keloids; (2) inability to receive treatment or follow‐up in accordance with regulatory requirements; (3) severe dysfunction of the cardiopulmonary, hepatic, or renal systems; (4) active tuberculosis or conditions that compromise the immune system; (5) hypersensitivity to corticosteroid preparations; (6) pregnancy, lactation, or planned conception; and (7) keloids located in the perineal or auricular regions.

### 2.3 Therapeutic Methods

#### 2.3.1 Stage I: Surgical and Perioperative Treatment

Procedures were conducted under intravenous or local anesthesia, depending on the size and location of the keloid. For local infiltration anesthesia, a 1:1 mixture of 2% lidocaine hydrochloride and 1:200000 epinephrine was used. Following anesthesia, a solution containing a 10:1 mixture of betamethasone (1 mL: 7 mg; comprising 5 mg betamethasone dipropionate and 2 mg betamethasone sodium phosphate; Hua Bang Pharmaceutical, Chongqing, China) and 5‐fluorouracil (5FU, 10 mL: 0.25 g; Jin Yao Pharmaceutical, Tianjin, China) was uniformly injected into the keloid until blanching was observed. Tissue multiple‐hole punches were performed using disposable skin biopsy punches (1, 2, 3, or 4 mm in diameter; Kai Industries, Tokyo, Japan) selected according to keloid thickness. For keloids 1–3 mm thick, 1–2 mm punches were used at 1–2 mm intervals. For those 3–5 mm thick, 2–3 mm punches at 2–3 mm spacing were recommended (Figure 2A). The punch was applied perpendicular to the skin surface and rotated down to the subcutaneous fat layer until the entire scar surface was densely drilled (Figure 2B). All excised tissue was removed, the wounds were irrigated with saline, and antibiotic ointment was applied. The wounds were then dressed with compression using sterile gauze. Superficial radiotherapy (SRT) was administered within 24 h postoperatively using the SRT‐100 device (Sensus Healthcare). Under the protection of a custom‐molded steel shield, patients received four fractions of SRT on consecutive postoperative days 0, 1, 2, and 3, with irradiation parameters as follows: 50 kV voltage, 15 cm from lesion to source, and a dose of 5Gy per fraction, for a total biologically effective dose of 20Gy per site.

#### 2.3.2 Stage II: Drug Injection

Drug injection was administered at 4 weeks following completion of Stage I. Patients were injected every 4 weeks for three consecutive injections. A 10:10:1 mixture of betamethasone, 2% lidocaine hydrochloride, and 5‐fluorouracil was injected intralesionally. Injections were performed using a 1 mL syringe and 30G needle, with approximately 0.05 mL/cm [2] injected at 0.5–1 cm intervals. For keloids presenting with localized pruritus, paresthesia, or protuberance, administer monthly intralesional injections until symptoms resolve without residual protuberance and the lesions approximate normal skin tone.

### 2.4 Evaluation Indices

The Patient and Observer Scar Assessment Scale (POSAS) and Vancouver Scar Scale (VSS) were used to evaluate the overall clinical efficacy of keloids at different observation time points. The observation points included pretreatment and days 30, 60, 90, 180, and 360 after the initial treatment. Clinical outcomes were assessed 12 months after the conclusion of Stage II. Follow‐up was conducted via clinic visits, telephone, or video consultations. The therapeutic response was classified as follows: (1) Marked Improvement: Lesion is entirely flattened and softened, color is white or approximating surrounding skin, and all symptoms (pain, pruritus) have resolved. (2) Improvement: Noticeable softening, incomplete flattening, mildly erythematous color; occasional itching or pain may remain. (3) Ineffective: No apparent change in keloid size, consistency, or symptoms. The severity of the worst lesion determined the therapeutic response.

Recurrence was defined as the reappearance of erythematous, indurated, and raised scar tissue at the site of a previously resolved lesion.

### 2.5 Adverse Events

The incidence of adverse events was recorded throughout treatment and follow‐up, including infection, localized ulceration or necrosis, telangiectasia, radiation dermatitis, skin atrophy, hyperpigmentation, hypopigmentation, and menstrual disturbances, among others.

## 3 Results

A total of 87 patients were enrolled in this study, including 36 females and 51 males, with an average age of (31.56 ± 12.28) years, ranging from 16 to 65 years. There were 55 married and 32 unmarried patients. The duration of keloid history ranged from 6 months to 41 years, with a mean duration of (6.89 ± 9.77) years. The anatomical distribution of keloids was as follows: Chest (20 cases), jaw (11 cases), shoulder (9 cases), and arm (5 cases). Multisite involvement was observed in several patients, with the lesions located at two sites in 13 patients, three sites in 16 patients, and four sites in 13 patients. In Stage II, patients received an average of (3.18 ± 0.58) treatments, with a maximum of six. At 12 months after completion of Stage II treatment, 78 patients (89.66%) achieved marked improvement, 7 patients (8.04%) showed improvement, and 2 patients (2.30%) were classified as ineffective. All patients completed the treatment protocol. The POSAS and VSS scores were recorded at pre‐treatment levels and at follow‐up points (30, 60, 90, 180, and 360 days) (Figure 1, POSAS and VSS scores pretreatment and after treatment at different time points). The POSAS and VSS scores decreased significantly during the follow‐up period. The two ineffective cases showed some early flattening after Stage I but had local ulcers that healed slowly. During their treatment, they experienced softening of the scar tissue and a reduction in symptoms. However, during the follow‐up treatment period, the scar tissue thickened and symptoms worsened again. No significant improvement in symptoms was observed by the end of Stage II treatment.

**FIGURE 1:**
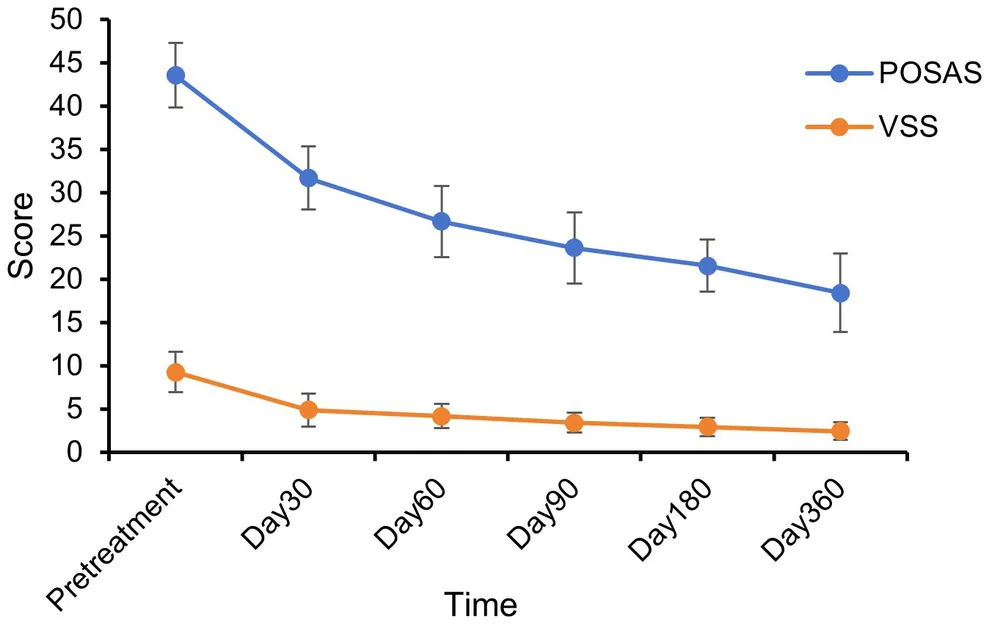
The POSAS and VSS scores pretreatment and after treatment at different time points.

### 3.1 Incidence of Adverse Reactions

All patients experienced varying degrees of hyperpigmentation within 2 months after the multiple‐hole punch excision procedure. Hyperpigmentation resolved in 80.75% of patients within 3 months after completion of Stage I treatment, with the longest duration lasting up to 12 months. During the Stage I treatment, four cases of localized ulcers and five instances of acute radiation dermatitis appeared. The localized ulcers, including two ineffective cases, healed after the dressing was changed. The patients with acute radiation dermatitis were all female and experienced relief after applying topical corticosteroid ointment. No other adverse reactions, such as infection, necrosis, telangiectasia, skin atrophy, or pigmentary changes outside of the above hyperpigmentation, were observed during treatment or follow‐up.

### 3.2 Typical Cases

Typical cases: We report two representative cases, as shown in Figures 2, 3, 4. All patients included in the study signed the informed consent.

**FIGURE 2:**
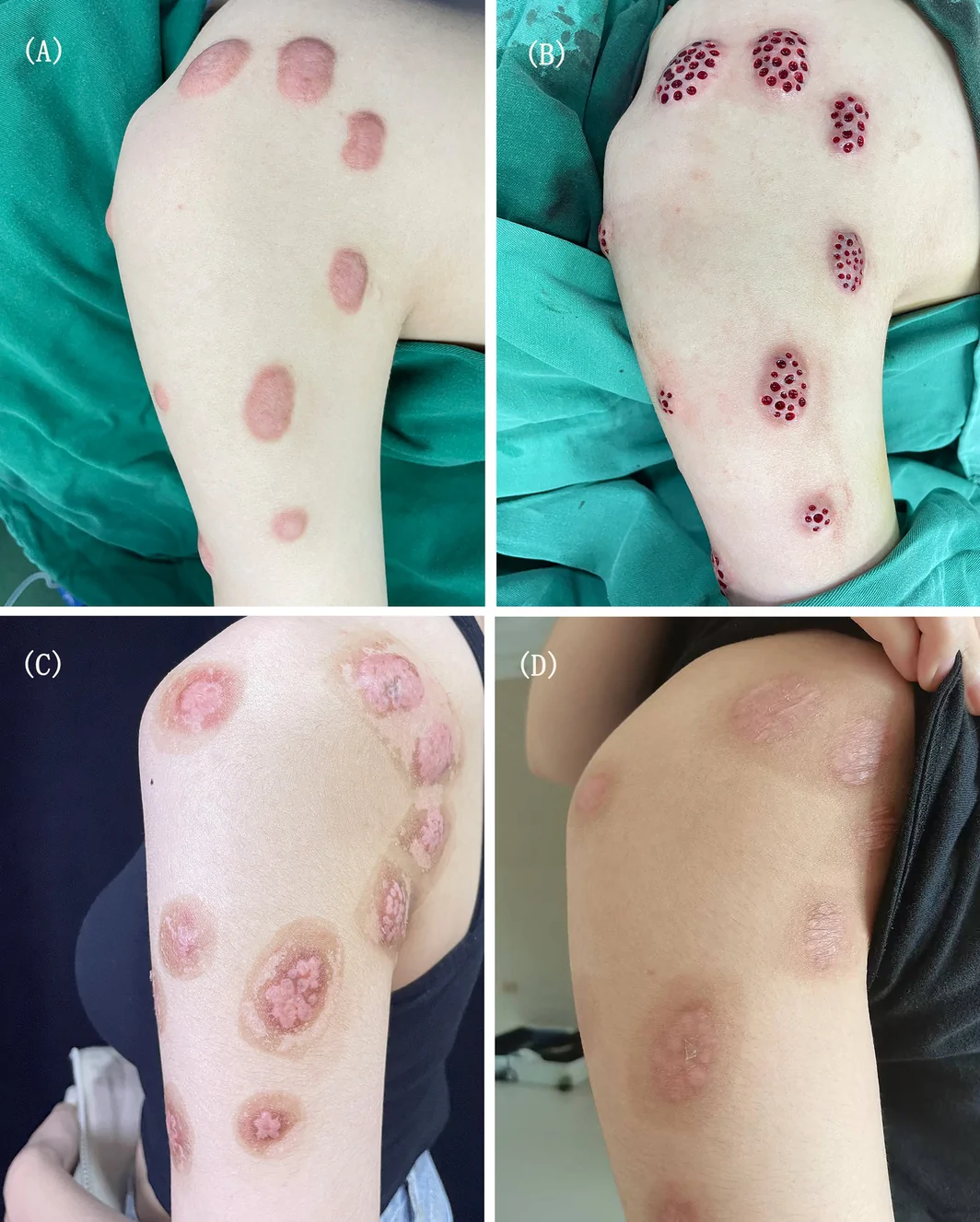
Patient A (A) before treatment. (B) The Immediate postoperative Stage I treatment with a multiple‐hole punch. (C) One month after Stage I treatment, SRT‐induced pigmentation can be observed. (D) Follow‐up was performed 6 months after completion of the Stage II treatment; the patient took a follow‐up photo at home.

**FIGURE 3:**
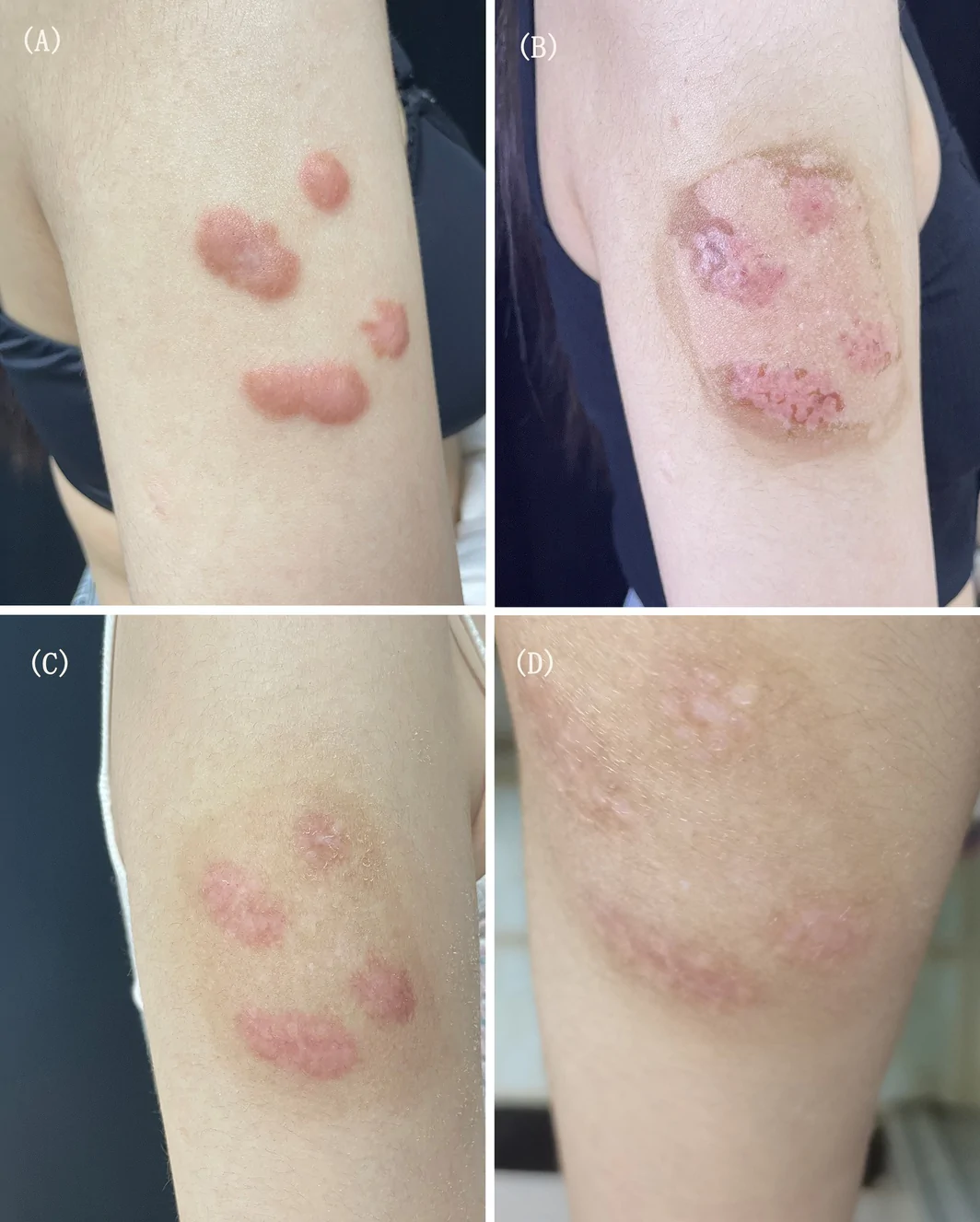
Patient B (A) before treatment. (B) The first treatment in Stage II. (C) End of the Stage II treatment. (D) Follow‐up was performed 12 months after completion of the Stage II treatment; the patient took a follow‐up photo at home.

**FIGURE 4:**
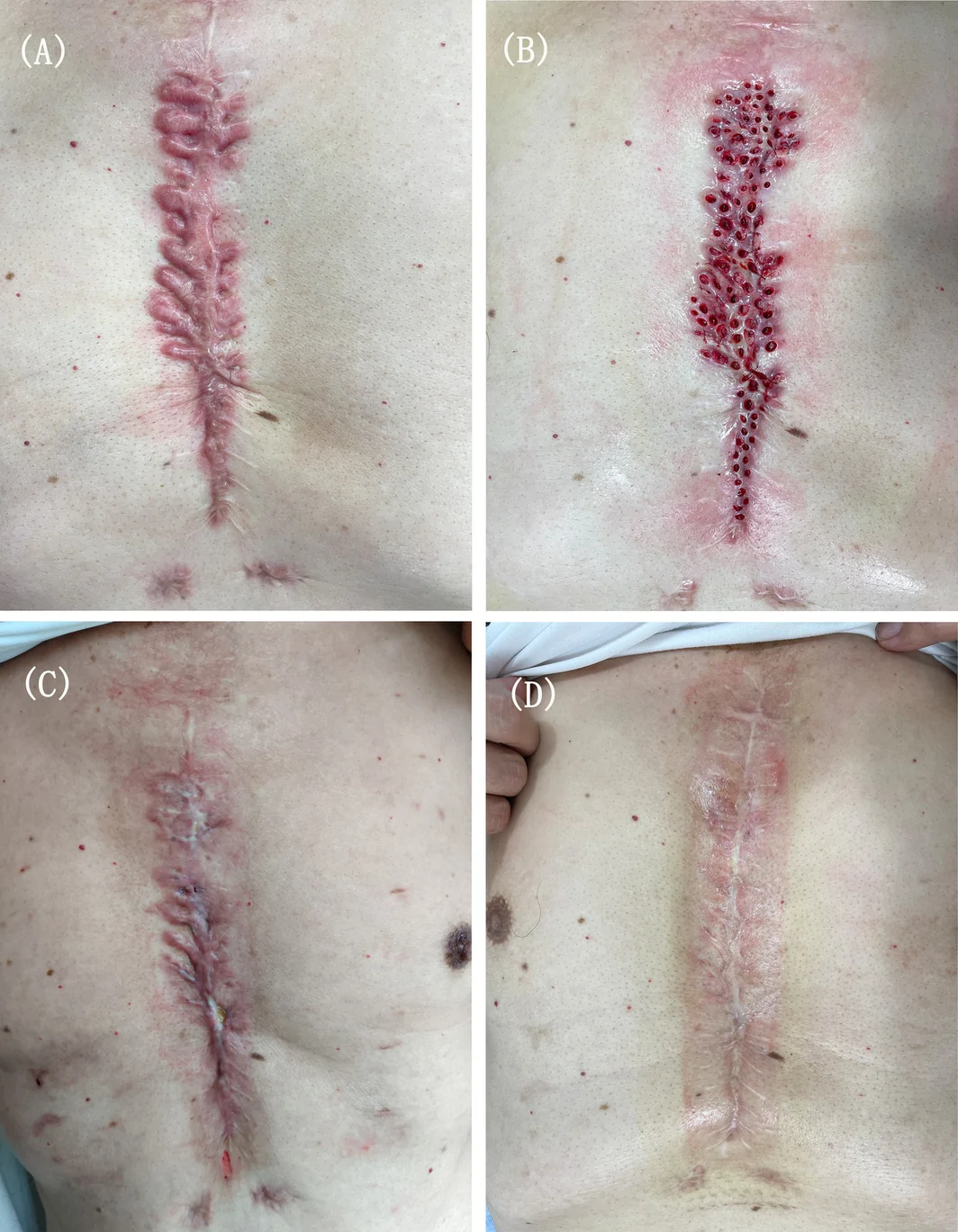
Patient C (A) Keloids on the chest following surgery for congenital heart disease. (B) The Immediate postoperative Stage I treatment with multiple‐hole punch. (C) Two months after Stage I treatment, local protrusions could be observed. (D) Follow‐up was performed 9 months after completion of the Stage II treatment.

## 4 Discussion

The management of dense and multiple keloids remains a significant therapeutic challenge due to their recalcitrant nature and high propensity for recurrence. Our sequential treatment—combining mechanical debulking via multiple‐hole punch excision, adjuvant superficial radiotherapy, and staged pharmacotherapy—demonstrated synergistic efficacy by targeting distinct pathological processes at different stages of keloid formation and recurrence. Three critical limitations of conventional approaches were addressed: (1) inadequate deep tissue penetration of intralesional agents, (2) mechanical tension perpetuation from bulk resection, and (3) sequential intervention against recurrence. In the initial phase, intralesional injection of a compound of betamethasone and 5‐fluorouracil (5FU) has multiple effects: (1) Following the injection of the drugs, the keloid becomes fuller and more rigid in texture, facilitating a more precise punch. (2) As an antineoplastic agent, 5FU acts as a radiosensitizer, enhancing the efficacy of subsequent superficial radiotherapy by increasing fibroblast radiosensitivity [6, 7]. Uppal's research has confirmed that wounds treated with 5‐fluorouracil produce scars with significantly lower levels of Ki‐67, vascular cell adhesion molecule‐1, and TGF‐β1 [8]. (3) The residual local presence of both drugs provides ongoing anti‐scar activity in the post‐procedure period, inhibiting excessive fibroblast activity. Intralesional corticosteroids are widely recognized as a mainstay of keloid therapy, and recent evidence supports increased efficacy when used in combination with other agents such as 5FU [9, 10, 11]. Our results align with this, demonstrating corresponding cure and improvement rates.

Importantly, minor trauma such as punch excision must be performed with great care in keloid patients, as even small injuries can trigger scar hypertrophy. Our experience highlights several technical considerations to optimize outcomes and minimize risk during punch excision:

- **Placement of the Punch:** The edge of the punch should be positioned precisely at the junction between the keloid and normal skin. This technique helps prevent injury to surrounding healthy tissue and minimizes the risk of further keloid enlargement.
- **Depth of Drilling:** The punch should be advanced as deeply as possible into the subcutaneous fat layer to facilitate complete removal of the tissue within the drill hole and maximize scar tissue excision.
- **Selection of Punch Diameter and Spacing:** The punch diameter and spacing should be tailored to keloid thickness. For keloids 1–3 mm thick, use a 1–2 mm punch with 1–2 mm intervals between holes. For keloids 3–5 mm thick, use a 2–3 mm punch with 2–3 mm intervals. Different diameters can be combined to maximize scar removal while preserving tissue blood supply.
- **Punch Orientation:** For thicker keloids, the punches are made at different angles. After creating a vertical punch, an additional punch is made at an angle through the same hole to remove more tissue.
- **Hemostasis:** To minimize intraoperative bleeding, which can interfere with the procedure, preoperative tumescence with local anesthetic solution should be performed around and beneath the keloid to reduce perioperative blood loss.
- **Exclusion of Excessively Thick Keloids:** Patients with keloids thicker than 5 mm were excluded from this study, as a single punch procedure is insufficient to flatten these lesions and multiple sessions would be necessary.

While multiple‐hole punch excision cannot completely remove all keloid tissue, it creates a mesh‐like structure that effectively reduces scar volume and internal mechanical tension, thereby lowering the risk of post‐procedural recurrence [12]. Notably, this approach presents fewer complications and is less invasive compared to complete surgical excision, making it accessible for implementation even in primary care settings.

Adjuvant radiotherapy for keloids represents a cornerstone for recurrence prevention [13]. Surgical excision alone is historically associated with recurrence rates as high as 45%–100%; combining surgery with adjuvant radiotherapy can reduce this to approximately 22% [14, 15], and postoperative electron beam radiotherapy has shown a recurrence rate similar to that of X‐ray radiotherapy [16]. In previous studies, the combination of punch and electron beam radiotherapy or superficial X‐ray therapy has demonstrated good efficacy in treating keloids [17, 18]. Electron beam radiotherapy has a long history of use and offers advantages such as controllable penetration depth and high precision. A substantial body of research has demonstrated the efficacy and safety of electron beam radiotherapy as an adjuvant treatment for preventing keloid recurrence following surgical excision [19]. However, in facilities lacking electron beam radiotherapy, superficial X‐ray therapy can serve as a viable alternative. Short treatment times, painless procedures, positive patient experiences, and high patient acceptance characterize superficial X‐ray therapy [18, 20]. It has been established that the application of relatively low total radiation doses generally does not result in skin and soft tissue reactions exceeding the common toxicity criteria (CTC) Grade 1–2 level [17]. Radiation therapy is associated with several common skin complications, such as erythema, desquamation, hyperpigmentation, hypopigmentation, and telangiectasia. In the cases studied, the main complications of radiation therapy were dermatitis, erythema, and hyperpigmentation; there were no instances of severe wound dehiscence or infection. Our study focused on patients with extensive, dense keloids not amenable to traditional surgery, showing that the combination of punch excision and timely SRT significantly improves outcomes by creating a wound environment more responsive to radiotherapy and inhibiting fibroblast proliferation, collagen synthesis, and neovascularization [16].

Multiple‐hole punch removes most of the scar tissue, and if there are signs of scar recurrence within the first three months postoperative, timely supplemental injections of the drug can help flatten the remaining scar tissue and reduce recurrence. The keloids have a relatively flat appearance after Stage I. However, the pathological structure of keloids has not changed substantially at this Stage [21]. To regulate their regrowth, drug injection was administered for one to three months following the punch intervention. In some patients, the local symptoms did not completely improve after three consecutive months of treatment in Stage II, and timely drug treatment could better control their further development. In this study, the maximum number of Stage II drug injections was six, and most patients were essentially stable after three consecutive treatments. This staged, cumulative approach leads to progressive reduction in keloid activity and improvement in clinical appearance. In terms of safety, hyperpigmentation was the most common adverse event, typically transient and resolving in most patients within three months postoperatively. In our study, both ineffective patients developed localized ulcers after the first phase of treatment. During their treatment, they experienced softening of the scar tissue and a reduction in symptoms. However, during the Stage II treatment period, the scar tissue thickened and symptoms worsened again. We believe these ulcers may affect the patients' overall prognosis. All of the patients who developed acute radiation dermatitis in our study were female, which aligns with the idea that individuals with darker skin tones are more prone to hyperpigmentation but less likely to experience acute radiodermatitis [22]. Our study ruled out keloids in the perineal and auricular regions, as keloids on the auricles of Asians are primarily caused by ear piercing. They are usually spherical or hemispherical and often thicker than 5 mm. Furthermore, infections frequently develop within the scar tissue. Punch excision fails to remove sufficient scar tissue, meaning it cannot completely release or eliminate the source of infection. As keloids in the perineal region often involve multiple hair follicles and are frequently accompanied by localized or widespread infection, punch excision cannot completely eradicate the source of the infection. Consequently, it is not the optimal treatment option for keloids in this area. There are two reasons for excluding keloids thicker than 5 mm. Firstly, the punch length is limited, so it would not be possible to reach the subcutaneous fat layer if the keloid were thicker than 5 mm. This would result in significant postoperative bleeding. Secondly, a considerable amount of scar tissue remains after the procedure if keloids are thicker than 5 mm, leading to suboptimal treatment outcomes. In our clinical practice, we use multiple punches for keloids thicker than 5 mm and only proceed with sequential treatment once effective volume reduction has been achieved.

Nevertheless, there are limitations to this study. The sample size was limited and derived from a single center, with no randomized control group. Due to the limited and unbalanced sizes across the three response subgroups (marked improvement, improvement, and ineffective), we did not perform statistical comparisons of baseline variables among these groups. The technique is less effective in cases of infected or contracted keloids. It may result in a longer healing period and more complex wound care compared to conventional excision or intralesional injections alone. We did not conduct a long‐term assessment of pigmentary abnormalities, nor did we use specialized equipment for objective monitoring.

Sequential treatment involving multiple‐hole punch excision, adjuvant SRT‐100, and pharmacotherapy demonstrates efficacy in managing multiple, dense keloids. This approach offers a relatively short treatment cycle, minimal invasiveness, and a low recurrence rate, providing an innovative and practical strategy for complex keloid management.

## Author Contributions

Y.A. developed the idea for the study and devised the methodology, while X.T. oversaw the revision of the article. Z.G. collected the data and wrote the manuscript, and W.J. analyzed the data and revised the manuscript. Z.Z. and W.R. were involved in providing cases, refining ideas, and discussing results. J.Y. and W.L. helped with data collection. Z.G., W.J, Z.Z., W.L., W.R., J.Y., X.T., and Y.A. All authors have read and approved the final manuscript.

## Funding

The authors have nothing to report.

## Ethics Statement

All procedures performed in studies involving human participants were in accordance with the ethical standards of the institutional research committee, batch number KY2023‐035‐01, and the ethical guidelines of the 1975 Declaration of Helsinki. All patients included in the study signed the informed consent, and the patients who offered images signed a photo consent statement.

## Conflicts of Interest

The authors declare no conflicts of interest.

## Data Availability

The data that support the findings of this study are available from the corresponding author upon reasonable request.
